# Regression tree construction by bootstrap: Model search for DRG-systems applied to Austrian health-data

**DOI:** 10.1186/1472-6947-10-9

**Published:** 2010-02-03

**Authors:** Thomas Grubinger, Conrad Kobel, Karl-Peter Pfeiffer

**Affiliations:** 1 Department of Medical Statistics, Informatics and Health Economics, Innsbruck Medical University, Schoepfstrasse 41/1, 6020 Innsbruck, Austria

## Abstract

**Background:**

DRG-systems are used to allocate resources fairly to hospitals based on their performance. Statistically, this allocation is based on simple rules that can be modeled with regression trees. However, the resulting models often have to be adjusted manually to be medically reasonable and ethical.

**Methods:**

Despite the possibility of manual, performance degenerating adaptations of the original model, alternative trees are systematically searched. The bootstrap-based method bumping is used to build diverse and accurate regression tree models for DRG-systems. A two-step model selection approach is proposed. First, a reasonable model complexity is chosen, based on statistical, medical and economical considerations. Second, a medically meaningful and accurate model is selected. An analysis of 8 data-sets from Austrian DRG-data is conducted and evaluated based on the possibility to produce diverse and accurate models for predefined tree complexities.

**Results:**

The best bootstrap-based trees offer increased predictive accuracy compared to the trees built by the CART algorithm. The analysis demonstrates that even for very small tree sizes, diverse models can be constructed being equally or even more accurate than the single model built by the standard CART algorithm.

**Conclusions:**

Bumping is a powerful tool to construct diverse and accurate regression trees, to be used as candidate models for DRG-systems. Furthermore, Bumping and the proposed model selection approach are also applicable to other medical decision and prognosis tasks.

## Background

The aim of *diagnosis related group (DRG) *systems is to classify hospital patients into clinically meaningful and comprehensible groups that consume similar hospital resources, usually measured by their *length of stay (LOS)*. These homogeneous patient groups are described by simple rules, often including the patients' diagnoses, procedures, sex and age. The aim of DRG is to use these parameters as an estimate for the resource consumption of the hospital's individual patients. Among other purposes, e.g. to monitor quality of care and utilization of services, one of their most important applications is a fair, performance-based allocation of available resources among hospitals.

Similar to the British *Healthcare Resource Groups (HRG) *[1] system and the Canadian *Case Mix groups (CMG) *[2] system, the Austrian DRG-system [3] is based on conjunctive rules only and no disjunctions are used, as is the case in other DRG-systems like the Australian *AR-DRG *[[4], Chapter H.3] and the German *G-DRG *[5] system. A major advantage of only using conjunctive rules is the possibility to interpret them as a tree structure, which gives a compact intuitively interpretable representation of the statistical model. Basically, these rules can be created by regression tree methods which, however, often have to be readjusted according to medical knowledge. Unfortunately, this manual adjustment usually yields a decrease of predictive accuracy.

Despite the possibility of manually adapting the original tree alternative models can be searched more systematically. One possibility for such and approach arises from an important characteristic of regression trees, i.e., their solutions are unstable. Thus minor changes in the data can result in completely different trees. Nevertheless, all of these trees can be statistically accurate. Through systematic resampling of the data by bootstrapping, a wider range of trees can be constructed. In this work, *bumping *[6] a bootstrap-based method proposed by Tibshirani and Knight is used.

In this article, we show that bumping allows us to build diverse and more accurate trees compared to the tree constructed by the currently used *Classification and Regression Trees (CART) *algorithm [7], while being equally or less complex. As it is shown in the results section, the statistically most accurate trees are too complex for the DRG-application. We propose to select the final models in a two-step approach from preprocessed models. In a first step the tree size is chosen based on the models' accuracies as well as economical and medical considerations. These considerations require a lot of domain knowledge and are very difficult to express numerically. Therefore, the final tree size can not be selected based on statistics alone, but has to be chosen manually. In a second step, given the pre-specified tree size, an accurate and medically reasonable model can be selected. In this way, statistically suboptimal, manual alterations of models are minimized.

### The Austrian DRG-System

Sine 1997 the Austrian hospital financing system is based on an activity-based hospital financing system called *Leistungsorientierte Krankenhausfinanzierung (LKF)*. The aim was to replace the beforehand used per diem-based payment scheme by a case-based one with following main objectives [8]:

• Consolidate rapidly increasing costs by reducing the LOS

• Reduce costs by substituting inpatient care through ambulatory care

• Make the hospital system more efficient

• Increase the transparency of costs and services

• Improve data quality

• Maintain the quality of medical services

• Ensure modern scientific methods in medical care

**Figure 1:**
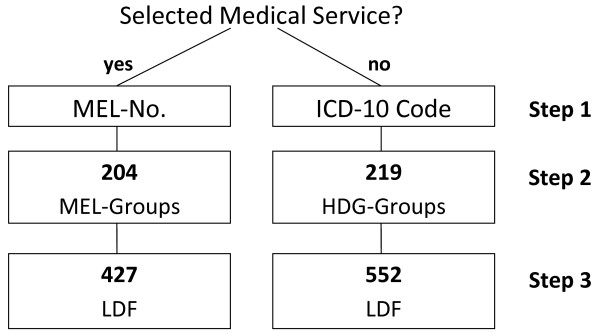
Overview of the three-step classification procedure of the Austrian DRG-system.

In the Austrian DRG-system in-hospital admissions are classified into homogeneous groups called *Leistungsorientierte Diagnosefallgruppen (LDF)*. The current model of 2009 is formed by a catalog of 979 patient groups resulting from a three-step classification procedure, summarized in Figure 1. First, the hospital patients are divided into two groups. In case a patient consumes a predefined individual medical service a procedure-oriented LDF, *Medizinische Einzelleistung (MEL)*, applies. Otherwise a LDF group related to the patient's main diagnosis, *Hautdiagnosegruppe (HDG)*, is selected. In the next step these two groups are clustered, based on their clinical similarity as well as on economical and statistical criteria, resulting in 204 MEL groups and 219 HDG groups. In the last step, patients corresponding to the MEL or HDG groups are further divided into 979 LDF, with the intention of finding groups with more homogeneous LOS. In this step, the patients' specific main diagnosis, secondary diagnoses, procedures, age and gender serve as possible split variables. The aim of using the LOS as the dependent variable is its good relationship with the total costs and its availability [8]. This final step of finding models to classify patients into the LDF groups is subject of this work and is displayed as *Step 3 *in Figure 1.

For the construction of the current LDF model the *CART *algorithm, a predictive tree model for regression and classification problems, was used. A main advantage of regression tree models is that they can be interpreted as simple rules without requiring any knowledge about the algorithm itself. This is particularly important as the final model is not only based on statistics, but its medical suitability also has to be evaluated by domain experts. For hospital management and budgeting these simple rules provide transparent information.

## Methods

### Regression Trees

The aim of regression tree analysis can be stated by explaining a continuous response variable *Y *by a vector of *n *predictor variables *X *= *X*_1_, *X*_2_,...,*X*_*n*_, which can be an arbitrary mix of continuous, ordinal and nominal variables. The CART algorithm recursively splits the data into two groups based on a splitting rule. The partitioning intends to increase the homogeneity of the two resulting subsets or nodes, based on the response variable. The partitioning stops when no splitting rule can improve the homogeneity of the nodes significantly.

**Figure 2:**
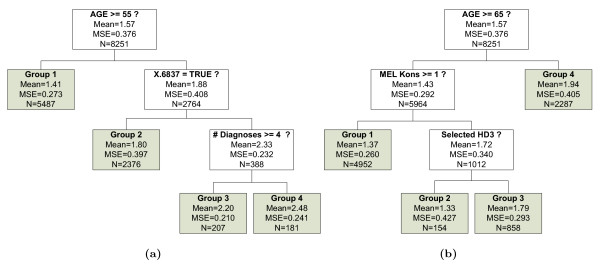
Two different trees constructed by bumping from the HDG0502 data. The two trees have different split points and variables, but have very similar predictive accuracy.

Splitting points are termed *internal nodes *and nodes without successors are called *terminal nodes*. A binary tree with *m *terminal nodes has *m *- 1 internal nodes. While the number of terminal nodes represents the number of patient groups in the model, the number of internal nodes can easily be interpreted as the required number of rules for classifying patients. In this paper the number of internal nodes is used as an measure of tree size or complexity, as for our application it is a more interesting interpretation than the total number of nodes. For regression problems the terminal nodes are formed by the averages of the response variables. The result can be represented by a tree structure, where nodes are connected via lines indicating the chain of recursive partitioning. Depending on the context, the terms *split *and *rule *are used throughout the manuscript, which, however both refer to what we have defined as a *splitting rule*. Two examples of a regression tree that determines the LDF group of the main diagnoses group HDG0502 are displayed in Figure 2.

The CART algorithm can be summarized by the following three steps [[7], Chapter 2]:

1. Examine every allowable split on each predictor variable. Commonly the binary splits are defined as *X*_*i *_<*c *for continuous variables and as *X*_*i *_∈ *C *for categorical variables, where *C *is a finite number of categories *b*_1_, *b*_2_,...,*b*_*m*_.

2. Select and execute the split that minimizes the impurity measure in the nodes. Samples that fulfill the criterion of the binary split propagate down into the descendant left node and the other variables into the right node. In our analysis we used the *least square *cost function, which is computationally efficient and the standard implementation of the CART algorithm.

3. Recursively continue step 1 and 2 on the descendant nodes until the homogeneity of the nodes cannot be improved significantly. Additionally, often additional stopping criteria are defined, e.g. minimum sample sizes in the terminal nodes.

Trees constructed in the described fashion tend to grow too big and have too few observations in the terminal nodes. In order to overcome this problem the trees are recursively pruned back to smaller size. In the DRG application we iteratively pruned back the internal node which led to the smallest degeneration in accuracy, until only one internal node remained. From there all tree sizes are evaluated separately.

Besides financial issues and medical preferences to split one HDG or MEL group further up than another, statistically the accuracy-complexity tradeoff of selecting the right sized tree can be assessed by the *cost-complexity *criterion defined as [[7], Chapter 3]:

where R(T) is the *Mean Squared Error (MSE) *and || is the number of terminal nodes, or the number of internal nodes minus one, of model T. *α *is a non-negative constant which regulates the additional cost for more complex trees.

### Requirements and Review of Alternative Tree Methods

There are many alternative regression tree algorithms, mainly differing by their tree structure, splitting criteria, pruning method and handling of missing values. In addition quite a lot of hybrid algorithms have been proposed, e.g. Quinlan's M5 algorithm [9] fits a linear regression model in each of the leaves to improve accuracy. Ensembles of trees [10] have become commonly used which are, on the other hand, less easy to interpret as the resulting model consists of more than one tree. Moreover, regression trees with soft splits [11] and methods to combine multiple trees into a single tree [12] were introduced. Both methods provide more accurate trees which, however, do not offer a distinct split point. Although, apart from the models accuracy, its low complexity, interpretability as well as its simple tree structure are most desirable properties for the DRG application.

The CART algorithm is a greedy algorithm which builds trees in a forward stepwise search. Therefore, its results are only locally optimal, as splits are chosen to maximize homogeneity at the next step only. By perturbing the data bumping identifies different trees in a greedy manner, while some of these models may be close to a global or local maximum. Besides the used bumping method, there are two other common groups of algorithms to find more globally optimal trees that fulfill our requirements of simplicity and interpretability, which are discussed in the following.

The first approach is to build trees in a globally more optimal way. This can be done by calculating the effects of the choice of the attribute deeper down in the tree, which in principle can be accomplished by an exhaustive search [13]. However, this is computationally intractable for larger data-sets. As a consequence, the search space is usually limited by heuristics. According to previous studies, look-ahead procedures are not always beneficial over greedy strategies and have been criticized [14,15]. On the contrary several authors [16-18] reported a significant improvement in tree quality. Murthy and Salzberg [14] conclude that limited look-ahead search on average produces shallower trees with the same classification accuracy. In some cases the trees from the look-ahead procedures are even both, less accurate and bigger than the trees produced by a greedy strategy. Quinlan and Cameron-Jones [15] argue that these rather unpromising results are due to oversearching the hypothesis space, resulting in an overfit of the training data.

Shi and Lyons-Weiler [19] presented the Clinical Decision Modeling System (CDMS), which allows searching for random classification trees that fulfill user specified constraints about model complexity and accuracy. Similar to our approach they follow the idea of constructing a set of models first and leave the selection of a clinically meaningful tree to the user of their software.

The second group of algorithms built the tree in a greedy manner first and improve the tree structure later by the use of optimization methods, e.g. evolutionary algorithms [20], Bayesian CART [21,22], simulated annealing [23] and tabu search [24].

Evolutionary algorithms are a family of algorithms that use stochastic optimization based on concepts of natural Darwinian evolution. For tree algorithms genetic operations can be applied to modify the tree structure and the tests that are applied in the internal nodes. Based on these operations new populations of trees are explored iteratively. The newly generated population is then assessed by a *fitness function*, which evaluates the quality of an individual within one population. Individual trees that are assessed to have a high fitness are more likely to be used in the next round, whereas the other models are rejected.

Kalles [25] classification tree algorithm uses a fitness function that takes the two quality attributes of misclassification rate and tree size into account. A survey of fitness approximations is given in [26]. An evolutionary approach that is applicable for classification and regression trees is presented in [20].

Bayesian CART [21,22] algorithms aim to stochastically optimize pre-specified CART trees in an approximated Bayesian way. The space of all possible trees is explored by Monte Carlo methods, which give an approximation to a probability distribution over the space of all possible trees. Modification of the tree structure is conducted by employing different move types, including *grow *and *prune *steps, as well as a *change *step which changes the split at an internal node. In contrast to evolutionary algorithms Bayesian CART is not population oriented, but only modifies one tree at a time.

Simulated annealing [23] is a stochastic search method that is inspired by the annealing of metals. An initial solution is modified by permutations and controlled by an evaluation function. Uphill moves, i.e., changes to a worse solution are accepted by the degree of badness and a parameter called *Temperature (T)*. When T is high the search is almost random, while at a lower temperature the updates are greedier. During the iteration T is slowly decreased and the time spent at a specific temperature is increased. The basic idea of simulated annealing is to avoid to get stuck in a local minimum to early when T is high and to find the local optimal solution when T is low.

From an initial tree model, tabu search [24] iteratively contacts several neighborhood moves, i.e., modifications of the tree, and selects the move with the best solution among all candidate moves for the current iteration. A set of admissible solutions is stored in a so called *candidate list*. The size of the candidate list determines the tradeoff between time and performance. Reversal moves are avoided by making selected attributes of moves *tabu*, i.e., forbidden. Tabu search allows searching for solutions beyond local optimum while still making the best possible move at each iteration.

### Model Search by Bootstrap

Bootstrap methods are most commonly based on the idea of combining and averaging models to reduce prediction error. Examples of such methods include Bagging [27], Boosting [28] and Random Forests [10]. The basic idea behind Bagging and Random Forests is to reduce variance by averaging a number of *B *models, created on the basis of *B *different data-sets. In contrast, Boosting reduces the overall training error by recursively fitting models to the residuals of the previously constructed regression tree. Although these methods can improve the accuracy and the variability of the results significantly, the final model itself loses its interpretability and the influence of the predictor variables becomes unclear.

In contrast to other bootstrap methods the result of bumping is not an ensemble of trees but only single trees, which are built on different bootstrap samples. The bootstrap samples themselves are formed by random sampling with replacement from the original training data, while each bootstrap sample has the same size as the original training data-set. This procedure is repeated *B *times, producing *B *bootstrap data-sets, from which, in turn *B *models can be built.

Bumping was successfully applied in combination with several learning algorithms including Classification Trees, Linear Regression, Splines and parametric density estimation [6], Linear Discriminant Analyis (LDA) [29], Neural Networks [30] and Self Organizing Maps (SOM) [31].

Tibshirani and Knight [6] selected the best tree regarding accuracy on the original training-set. In our application the best *j *trees for each tree complexity, measured by the number of internal nodes, are of interest. This is because we want to construct different models first and leave the decision about the final model to medical domain experts. The bumping procedure can be summarized as follows:

1. A set of bootstrap samples z^*1^, z^*2^,...,z^*B ^are drawn from the training-set *z*

2. Models are fit to each bootstrap sample giving prediction (*x*) for each bootstrap *b *= 1, 2,...,*B *at input point *x*. As a convention the original training-set *z *is included among the B bootstrap samples as well.

3. For each tree complexity, the best trees are selected based on their average prediction error on the original training-set *z*.

In the following section the evaluation of the selected trees on independent data is further discussed. Additionally, the evaluation criteria to assess the number of statistically accurate model choices are defined.

From the presented methods that allow searching for alternative tree models, only bumping and evolutionary algorithms offer a diverse set of model choices. However, in principle the other methods could be modified to store an arbitrary amount of accurate candidate trees that are created during the search process.

A particular advantage of bumping compared to other non-greedy regression tree methods is the possibility to computationally effective construct and select the best models for each tree size. By the use of bumping all candidate trees can simply be grown to full size first and secondly be pruned back iteratively by one node. As a result, for each tree size the best model can be selected from the *B *bootstrap trees. Other algorithms that search for globally optimal candidate models would tend towards trees that are optimal for some tree complexity. These trees would either be very complex, or would at least have similar complexity for all candidate trees if the models' quality is measured by accuracy and the complexity of the tree. However, iterative pruning of these models does not necessarily result in optimal models with smaller tree size. Therefore, in order to build optimal trees for each tree size, each model complexity, determined by the number of internal nodes, would have to be handled separately.

For look-ahead algorithms this computational requirements would be very high as binary trees with a given number of nodes can have different forms. That is, there are many possible arrangements, called topologies, for a given number of internal nodes. Therefore, for each tree complexity a number of topologies *C*_*n *_would have to be considered. Where *C*_*n *_grows exponentially with the number of nodes *n *and is given by the Catalan number [32]:

where *C*_*n *_is the number of topologies for trees with *n *internal nodes. The number of binary trees with *n *= 1 to *n *= 6 internal nodes are 1, 2, 5, 14, 42, 132.

### Evaluation Criteria

The performance of bumping compared to the standard CART algorithm is evaluated based on its ability of finding homogeneous patient groups with similar LOS. That is modeling and predicting the LOS of hospital patients, as it is described in the third step of the three-step classification procedure, summarized in Figure 1.

**Figure 3:**
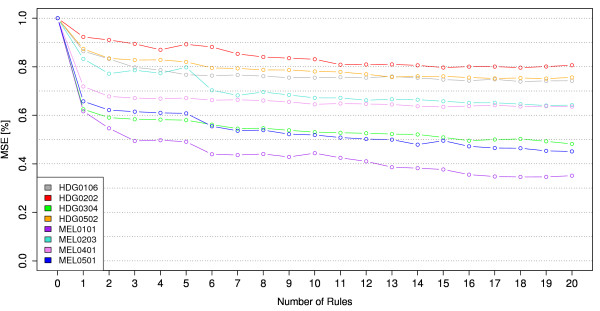
Reduction of the MSE obtained by the best bootstrapped tree for different tree sizes.

Tree size has a big influence on the accuracy of models. Since bumping compares different models on the training data, the models must have similar complexity [[7], Chapter 8], given by the number of internal nodes. Therefore, only models with the same number of nodes are compared in our analysis. We limited model sizes to a maximum number of 16 internal nodes resulting in a maximum of 17 patient groups and a tree depth of 5 corresponding to a maximum of 5 rules to classify patients. As it can be seen in Figure 3 more complex models only gave relatively small improvements in predictive error and were considered as too complex for our application. As a comparison, in the LKF model 2009 the most complex tree has 11 internal nodes and few trees have more than 4 internal nodes. For each comparison *B *= 200 bootstrap samples were drawn from the training-set. We used the R package *rpart *[33] to build regression trees. The suitability of the bootstrap method is evaluated in two ways, which are described in the following.

#### Accuracy of the Best Bootstrapped Tree

In this first evaluation step we want to show that the best bootstrapped tree offers increased predictive accuracy compared to the CART algorithm. The difference in accuracy is assessed by the use of *10-fold cross-validation *[[34], Chapter 7]. In 10-fold cross-validation the data is first partitioned into complementary subsets called folds. The model is then built on 9 folds and the remaining fold is used as a test-set. This analysis is repeated 10 times, where each of the folds is used as the test-set once. Finally, the estimate of predictive accuracy is calculated from the average performance of the 10 models on their associated test-sets. The evaluation on independent data is especially important as a wider search of the hypothesis space can lead to overfitting of data [15].

To avoid overfitting, each terminal node should have a minimal amount of observations *m*_*min*_. However, in our comparison, we did not restrict the minimum number of *m*_*min*_. The reason is, that we want to avoid the effect of trees stopping to split with *m*_*min *_- *k *observations, where *k *is a small number of instances, while similar trees with *m*_*min *_observations further split up. To give an example where this is important imagine that the standard CART tree stops splitting at node *j *with *m*_*min *_- 1 nodes. One of the 200 bootstrap trees is very similar to the standard CART tree but has *m*_*min *_observations in node *j*. As a result the bootstrap tree splits at *j *while the CART tree stops splitting. Thus, this marginal difference of one more observation in *j *results in two different tree-topologies which can have significantly different predictive accuracy.

#### Number of Accurate Model Choices

In the second step of our evaluation the possibility to construct diverse choices of accurate trees by the use of bootstrap sampling is presented. The estimation of accuracy takes the whole data-set into account. In this part of the evaluation, where we assess the number of diverse choices of accurate trees, we limited the minimum number of observations to 30, which we thought of to be large enough to avoid overfitting as well as to be a minimum requirement to form a patient group in the LKF model.

### The DRG-Data

The basis for our analysis are 8 data-sets, 4 MEL and 4 HDG groups of the Austrian DRG-system 2006. The data-sets consist of information about the patients' main diagnosis, secondary diagnoses, procedures, number of diagnoses, number of procedures, sex and age, as well as the patients' length of stay. The characteristics and a short description of the medical meaning of the evaluated data-sets are summarized in Table 1. Permission to use the data was granted by the *Bundesministerium für Gesundheit, Familie und Jugend (BMG) *[35].

**Table 1:** Description of the evaluated data-sets.

Data-Set	Description	Sample Size	Variables (Interval,Nominal)	
HDG0106	Parkinson's disease	6155	114	(109,5)
HDG0202	Malignant neoplasms	3933	55	(47,8)
HDG0304	Eye diagnoses	9067	41	(36,5)
HDG0502	Acute affections of the respiratory tract and middle atelectasis	8251	100	(92,8)
MEL0101	Interventions on the skull	875	60	(54,6)
MEL0203	Small interventions in connective tissue and soft tissue	17268	58	(52,6)
MEL0401	Interventions on the outer and middle ear, designed to treat a liquorrhoe	4102	44	(40,4)
MEL0501	Interventions on the esophagus, stomach and diaphragm	3432	86	(80,6)

## Results

### Accuracy of the Best Bootstrapped Tree

**Figure 4:**
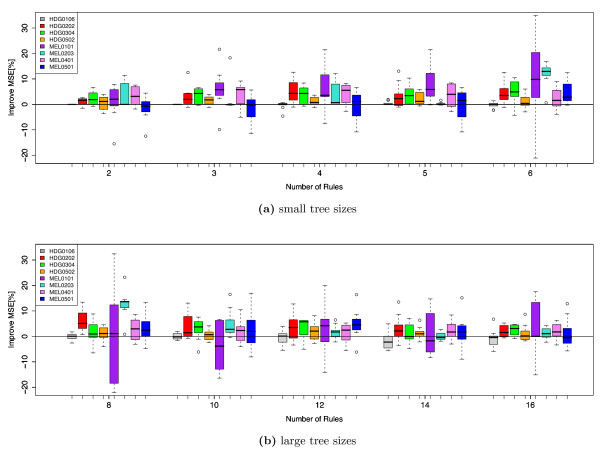
Comparison of the best bootstrap based tree with the standard CART tree.

Results of the relative predictive accuracy of the best bootstrapped tree compared to the CART tree are displayed in Figure 4. The individual Boxplots refer to one data-set and one possible tree complexity each and result from the 10 test-sets from the cross-validation procedure. Although the bootstrap based trees are not always better than the standard CART trees, it can be observed that on average they offer increased accuracy for most data-sets and tree complexities. The high variability of the relative performance is also due to a relative small portion of test-data (10%) of each fold. However, as each observation serves as a test-sample in one of the folds, the average of the results gives a good estimate of the predictive accuracy. No obvious relationship between the variability of the relative performance and the complexity of the trees can be observed.

Table 2 summarizes the results in less detail by displaying the average change in relative accuracy for each data-set and tree size. It can be observed that for 5 of the 8 data-sets the average accuracy improved for all evaluated tree sizes. The HDG0106 main diagnosis data-set is the only one where the best bootstrap trees performed worse than the standard CART trees. The bootstrap method also performed worse for models with small tree sizes (2-4 internal nodes) for the MEL0501 data-set as well as for trees with 10 and 14 internal nodes for the MEL0101 data-set. However, the majority of the bootstrap trees outperformed the standard CART trees. Averaged over all data-sets an improvement of 1.06-4.90% for the different tree sizes could be achieved. No specific reason for the worse performance, of the bumping methodon the two data-sets HDG0106 and MEL0501 could be found.

**Table 2:** Relative average improvement.

Tree Size	HDG0106	HDG0202	HDG0304	HDG0502	MEL0101	MEL0203	MEL0401	MEL0501	Average
2	0.00	1.12	2.55	0.71	1.20	3.74	3.34	-1.52	**1.39**
3	0.00	2.78	3.33	1.65	5.96	1.88	3.92	-1.97	**2.19**
4	-0.36	5.57	3.52	1.23	5.77	3.30	4.28	-1.05	**2.78**
5	0.42	3.18	3.85	2.30	7.43	0.26	3.81	-0.84	**2.55**
6	-0.24	4.38	5.47	1.13	9.65	12.03	2.33	4.41	**4.90**
8	-0.11	6.05	1.75	1.15	1.06	12.91	2.67	3.63	**3.64**
10	-0.06	3.99	3.16	0.69	-2.93	5.09	1.94	2.83	**1.84**
12	-0.42	4.14	3.24	1.75	2.89	1.61	1.24	4.95	**2.43**
14	-1.87	3.35	1.82	1.20	-0.36	0.00	2.15	2.17	**1.06**
16	-0.76	2.11	2.52	1.27	1.38	1.18	1.89	0.65	**1.28**

Relative average improvement of the best bootstrapped tree compared to the standard CART tree using 10-fold cross validation.

Figure 3 illustrates the reduction of the total MSE by models with different tree complexities estimated by 10-fold cross validation. It can be observed that the predictive error is already reduced with a small number of splits and the improvements obtained by additional splits become progressively smaller with increasing tree complexity. Although very large trees often give the best predictive performance, these complex trees are difficult to interpret and hard to work with.

The average improvement in relative accuracy by the bootstrap method often offers models with the same accuracy but less complex rules. For example, models with 3 internal nodes compared to models with 2 internal nodes offer an average increase in accuracy of 1.60%, while the accuracy of the bootstrap method achieved an average improvement of 1.39%. For the data-sets HDG0304, MEL0203 and MEL0401 the best bootstrapped tree with 2 internal nodes even outperforms the CART tree with 3 internal nodes. This effect becomes even more significant for larger tree sizes where one or even several rules can be omitted without degeneration in performance.

### Number of Accurate Model Choices

In the second step the number of trees constructed by bumping that are at least as accurate or better than the standard tree is evaluated. Models are considered dissimilar when at least one split variable differed between the trees. For groups of trees where all the split variables are the same, but the split points differ the most accurate tree is selected and considered as a candidate model.

In Table 3 the numbers of distinct accurate trees are broken down into accuracy classes for each tree complexity. The results are displayed as the mean, minimum and maximum number of different trees constructed on the 8 evaluated data-sets and are within an accuracy class. To give an example, for models with 4 internal nodes on average 23.3 distinct trees with a minimum performance in relative accuracy of *-*1% were constructed. The minimum number of distinct trees constructed on one of the data-sets is 6 and the maximum number is 67. From these 23.3 different models an average of 7.4 trees have a relative improvement of accuracy > 1% and in turn 3.9 trees achieved a relative improvement of > 3%.

**Table 3:** Number of diverse trees.

Tree Size	[-1	%, ∞]	[+1	%, ∞]	[+3	%, ∞]
2	3.4	(0,9)	0.1	(0,1)	0.1	(0,1)
3	14.1	(0,45)	4.8	(0,27)	1.3	(0,9)
4	23.3	(6,67)	7.4	(0,23)	3.9	(0,21)
5	30.3	(10,45)	12.4	(0,37)	7.1	(0,34)
6	39.8	(7,66)	10.4	(0,47)	4.3	(0,34)
8	42.9	(12,84)	2.5	(0,8)	0.0	(0,0)
10	60.1	(10,115)	9.0	(0,29)	0.1	(0,1)
12	63.4	(6,181)	12.1	(0,93)	0.0	(0,0)
14	76.1	(5,183)	13.1	(0,70)	8.8	(0,70)
16	82.5	(5,187)	16.6	(0,98)	1.9	(0,15)

Number of diverse trees with an improvement in relative accuracy of [*min*%*, max*%] compared to the CART tree, displayed as *mean*(*min, max*) referring to the mean, minimum and maximum number of trees constructed on the 8 evaluated data-sets.

The results show that even for very low tree complexities alternative models can be found. For simplest models, with only 2 internal nodes, an average of 3.4 different trees with at least similar accuracy [-1%, +1%] were found. For slightly more complex models with 3 rules the average number of models with at least similar accuracy increased to 14.1 and 4.8 trees offered improve accuracy of > 1%, compared to the standard CART tree. It can be observed that with increasing model size the number of different trees increases to 187 for models with 16 internal nodes, while many of these models only differ by minor important splits at the bottom of the trees, which do not contribute much to the reduction of impurity and are medically very similar.

Therefore the similarity of trees should be further distinguished. How to assess statistical similarity of trees by means of topography and similar partitioning is discussed in [36,37]. However, in the DRG-application we are mainly interested in the choices of split-variables regarding their medical meaning. In our analyses nodes differing further up in the tree are considered as more influential, as more patients are affected by these rules and they also contribute more to the reduction of the total variance. As an estimate on which levels the differences occur the results from Table 3 can be taken into account.

## Conclusions

Based on the evaluation of 8 large data-sets taken from the Austrian DRG system, we showed that bumping can be used to construct diverse and accurate candidate models for DRG-systems that are based on conjunctive rules. Compared to other methods that allow a broader search of the hypothesis space, bumping can be used computationally more efficient. The presented results show that on average the predictive accuracy of the best bootstrap based tree offers improved accuracy compared to the tree from the standard CART algorithm. Furthermore, less complex trees can be found that are non-inferior compared to the single tree constructed by the original algorithm.

During the whole development of the Austrian DRG-system medical experts have been involved in the evaluation of the resulting regression trees. Many times the statistical optimal tree was not selected because of medical expert opinion. From discussions with medical experts, we know that a single, data-driven model is not always the medical correct one and different options have to be presented for medical evaluation. With our approach of constructing diverse models for different pre-specified tree sizes, we allow a wide range of candidate models to be considered. For these candidate models suitable tree sizes can be selected, based on the cost-complexity criterion as well as on economical and medical considerations. Subsequently, given a desired tree complexity, medical domain experts can chose a final model. In this way, statistically suboptimal, manual alterations of models can be minimized.

This presentation illustrates the possibilities of bumping, which will be used in the next years of the maintenance and further development of the Austrian DRG-system. Besides its relevance to DRG-systems, bumping and the proposed two-step model selection process are especially useful to assist in any kind of classification or regression problems in medical decision and prognosis tasks [38-40]. This is because domain specific knowledge can be used to guide the selection of a medically meaningful and statistically accurate model.

## Competing interests

The authors declare that they have no competing interests.

## Authors' contributions

TG is the main author of this article. He participated in the design of the study, conducted the experiments and drafted the manuscript. CK provided knowledge about DRG-systems and helped to draft and review the manuscript. Since 1991, KPP is one of the project managers for the development of the Austrian LKF system. He participated in the study with his expert knowledge about DRG-systems, provided the data, contributed in the design of the study and reviewed the manuscript. All authors read and approved the final manuscript.

## Pre-publication history

The pre-publication history for this paper can be accessed here:

http://www.biomedcentral.com/1472-6947/10/9/prepub
